# Supplementary material for: Genome duplication in Leishmania major relies on persistent subtelomeric DNA replication
Source: eLife. 2020 Sep 8;9:e58030. doi: 10.7554/eLife.58030 (PMC7511235; doi:10.7554/eLife.58030)
Supplement: Transparent reporting form [file elife-58030-transrepform.docx]

***eLife’s* transparent reporting form**

We encourage authors to provide detailed information *within their submission* to facilitate the interpretation and replication of experiments. Authors can upload supporting documentation to indicate the use of appropriate reporting guidelines for health-related research (see [EQUATOR Network](http://www.equator-network.org/%20)), life science research (see the [BioSharing Information Resource](https://biosharing.org/)), or the [ARRIVE guidelines](http://www.plosbiology.org/article/info:doi/10.1371/journal.pbio.1000412) for reporting work involving animal research. Where applicable, authors should refer to any relevant reporting standards documents in this form.

If you have any questions, please consult our Journal Policies and/or contact us: [editorial@elifesciences.org](mailto:editorial@elifesciences.org).

**Sample-size estimation**

- You should state whether an appropriate sample size was computed when the study was being designed
- You should state the statistical method of sample size computation and any required assumptions
- If no explicit power analysis was used, you should describe how you decided what sample (replicate) size (number) to use

Please outline where this information can be found within the submission (e.g., sections or figure legends), or explain why this information doesn’t apply to your submission:

Flow cytometry, cell sorting and imaging were performed according to published guidelines.

All genome-wide analysis, including MFA-seq, were performed using publicly available bioinformatic tools and previously published standards.

References and approaches provided in methods.

**Replicates**

- You should report how often each experiment was performed
- You should include a definition of biological versus technical replication
- The data obtained should be provided and sufficient information should be provided to indicate the number of independent biological and/or technical replicates
- If you encountered any outliers, you should describe how these were handled
- Criteria for exclusion/inclusion of data should be clearly stated
- High-throughput sequence data should be uploaded before submission, with a private link for reviewers provided (these are available from both GEO and ArrayExpress)

Please outline where this information can be found within the submission (e.g., sections or figure legends), or explain why this information doesn’t apply to your submission:

Biological replicates: experiments conducted on separate occasions, using independently grown parasite cell cultures.

Technical replicates: evaluation of effect from single biological replicate evaluated by separate experiments (e.g. independent aliquots from one sample).

Fig.1. A: 3 biological replicates. B: 2 biological replicates. C: 2 biological replicates with >200 cells counted for each one.

Fig. 1 – Fig. 7. MFA-seq across these figures was performed in at least five biological replicates, including with two wild type strains, two mutants and HU treatment.

**Statistical reporting**

- Statistical analysis methods should be described and justified
- Raw data should be presented in figures whenever informative to do so (typically when N per group is less than 10)
- For each experiment, you should identify the statistical tests used, exact values of N, definitions of center, methods of multiple test correction, and dispersion and precision measures (e.g., mean, median, SD, SEM, confidence intervals; and, for the major substantive results, a measure of effect size (e.g., Pearson's r, Cohen's d)
- Report exact p-values wherever possible alongside the summary statistics and 95% confidence intervals. These should be reported for all key questions and not only when the p-value is less than 0.05.

Please outline where this information can be found within the submission (e.g., sections or figure legends), or explain why this information doesn’t apply to your submission:

In all metaplots throughout the main and supplementary figures, lines are means and shaded areas around them are SEMs.

Number of regions considered in each metaplot are as follows: AcH3/KKT1/BaseJ, 35; AcH3/BaseJ, 47; AcH3 Intern, 38 and AcH3 End, 30.

Number of regions considered in each heatmap is 36.

Units used in metaplots, heatmaps and boxplots are stated in each of them.

(For large datasets, or papers with a very large number of statistical tests, you may upload a single table file with tests, Ns, etc., with reference to sections in the manuscript.)

**Group allocation**

- Indicate how samples were allocated into experimental groups (in the case of clinical studies, please specify allocation to treatment method); if randomization was used, please also state if restricted randomization was applied
- Indicate if masking was used during group allocation, data collection and/or data analysis

Please outline where this information can be found within the submission (e.g., sections or figure legends), or explain why this information doesn’t apply to your submission:

Do not apply.

**Additional data files (“source data”)**

- We encourage you to upload relevant additional data files, such as numerical data that are represented as a graph in a figure, or as a summary table
- Where provided, these should be in the most useful format, and they can be uploaded as “Source data” files linked to a main figure or table
- Include model definition files including the full list of parameters used
- Include code used for data analysis (e.g., R, MatLab)
- Avoid stating that data files are “available upon request”

Please indicate the figures or tables for which source data files have been provided:

Sequences used in this study have been deposited in the European Nucleotide Archive. Data can be accessed using the accession number PRJEB35027.
